# Dietary Supplements: Are Current Policies Adequate for Promoting Health?

**DOI:** 10.3390/nu12113449

**Published:** 2020-11-11

**Authors:** Zumin Shi, Alice Yan

**Affiliations:** 1 Human Nutrition Department, College of Health Sciences, QU Health, Qatar University, Doha 2713, Qatar; 2 Center for Advancing Population Science, Department of Medicine, Division of General Internal Medicine, Medical College of Wisconsin, Milwaukee, WI 53226, USA; aliceyan@mcw.edu

Globally, there has been a dramatic increase in the use of dietary supplements over recent decades. It is estimated that the supplement market will reach USD 300 billion in the next 5 years [1]. The market may become even larger due to the COVID-19 pandemic. A recent online Council for Responsible Nutrition (CRN) COVID-19-focused consumer survey of 2400 adults aged 18 years and older living in the United States discovered that more than two in five (43%) dietary supplement users have changed their regiments during the COVID-19 pandemic in a way that boosts their overall usage [2]. The top two reasons supplement users increased their supplement routine were for “overall immune support” and for “health/wellness benefits”. In an online survey conducted in China during the COVID-19 pandemic, one third of the participants reported using dietary supplements or Chinese herbs [3]. Dietary supplements are a lucrative industry for many reasons, including the high demand for the products and the relatively low cost to develop the products. Furthermore, unlike drugs, consumers do not always expect to have an immediate effect upon consuming the products. Due to the high use of dietary supplements and the potential health benefits as well as adverse effects, there is an urgent need for better regulation. 

In their review article, Dwyer J.T. et al. comprehensively examined the challenges in the use, regulation, and research of dietary supplements [4]. Based on their review, one of the challenges is that there is no consensus on the definition of dietary supplements internationally. It varies between nutritional supplements, herbal medicines and traditional medicines. Regulations are different from country to country. However, it is clear that the regulations on dietary supplements in different countries are much less stringent than those of prescribed or over-the-counter medicines. The review also presented a case study and highlighted some examples of research supported by the Office of Dietary Supplements (ODS), National Institutes of Health (NIH), USA. The Dietary Supplement Label Database created by the ODS contains over 72,000 products sold in the USA, which indicates the scale of the workload to regulate these products.

Dietary supplements are necessary for specific groups of people. For example, vegetarians are required to have vitamin B12 supplementation and people with iron deficiency need to have iron supplementation. In recent history, nutrient supplements have contributed substantially to human health.

The efficacy of dietary supplements remains a central focus in research. The gold standard to test the efficacy of a dietary supplement is through a randomized clinical trial (RCT). RCTs are often conducted in a selected population, do not have sufficient duration of follow-up and have a limited number of hard outcome events. Findings from the limited available RCTs are unlikely to be generalized. More research is needed before solid conclusions can be made for most dietary supplements. Even for omega-3s, the most-studied supplements, their health effects remain uncertain [5]. The effect of dietary supplement use in the general population is not the same as we expected. The latest systematic reviews and meta-analyses of the most common dietary supplements yielded disappointing findings. For example, the latest meta-analysis of clinical trials and prospective cohort studies showed that multivitamin/mineral (MVM) supplementation does not improve cardiovascular outcomes in the general population [1]. 

For most consumers, one of the aims of taking dietary supplements is to increase longevity. However, data from the 1999–2010 National Health and Nutrition Examination Survey (NHANES) suggest that supplement use does not reduce the risk of mortality in the general population [6]. Taking more dietary supplements than you need costs more and might also elevate the risks of side effects. For example, excess intake of calcium (i.e., taking in at least 1000 milligrams of calcium a day from supplements) is associated with a higher risk of cancer death, according to a recent study published in Annals of Internal Medicine [6]. Some of the most commonly used dietary supplements are herbal products. The interaction between herbal products and medication is well documented, especially in patients with cardiovascular diseases [7]. However, physicians are not often aware of their patients’ use of these products. What is more worrisome is that patients may use herbal products and not follow formal medical treatment. This is particularly the case in Asia, where the use of traditional herbal medicine is common. For example, in China, functional foods are accredited and allowed to advertise their functions. This puts some people at risk because it is difficult for some consumers to distinguish the difference between a functional food and a drug. Unlike drugs, the adverse effects of dietary supplements, including herbal products, are not systematically reported and monitored. In fact, RCTs on herbal products with long follow-up durations are rare. Herbal supplements and multivitamin and multimineral use are common in children but studies on the longer-term effects are limited. Even the more commonly used nutrients and vitamin supplements are not necessarily risk-free. For example, based on the Alpha-Tocopherol, Beta-Carotene Cancer Prevention (ATBC) study and the Beta-Carotene and Retinol Efficacy Trial (CARET), beta-carotene supplements of 20 or 30 mg/day increase the risk of lung cancer among smokers [8,9]. 

If scientists, governments and industries do not have a clear consensus on the regulations of dietary supplements, how can consumers make the right choices? Taking melatonin as an example, as pointed out by Dwyer J.T. et al., it is treated as a dietary supplement in the USA but regulated as a prescription medicine in Australia [4]. In China, melatonin is approved as a functional food. The main ingredient of one of the most popular health supplements, named “Nao Bai Jing,” is melatonin. It is widely advertised as a panacea for brain health in China and many consumers purchase it as a gift. Consumers need valid, scientifically-based information on dietary supplements to guide their consumption rather than advertisements from dietary supplement companies. In many countries, the health-food market is largely driven by advertisements. Although these food advertisements are regulated, violation of the regulations is not uncommon. Dietary guidelines are available in most countries. However, there is no guideline on dietary supplement use for the general public. Dietary supplement guidelines should be specific to the population as the dietary intake and nutritional status are different in different countries. The need for dietary supplements may depend on the diet and disease spectrum. For example, several decades ago, iron deficiency anemia was a major health problem in many developing countries. Iron supplementation was needed to prevent anemia. However, with economic development and dietary change in these countries, the prevalence of anemia decreased substantially [10]. The need for iron supplementation in the general population does not exist. In contrast, high iron intake has been shown to increase the risk of diabetes, hyperuricemia and cognitive impairment among adults in China [11,12,13]. Furthermore, this detrimental effect of iron is stronger among those with obesity [11].

A few studies have been conducted to assess the attitudes and beliefs of dietary supplements among consumers and health professionals [14]. It is worrisome that the majority of the consumers did not discuss their use of supplements with their physicians.

Establishing methods for testing bioactive components and conducting clinical trials are necessary to ensure the safety and efficacy of dietary supplements. A substantial amount of funding has been put toward dietary supplement-related research by the NIH, with a total of USD 855 million between 2009 and 2011 [15]. However, even if some supplements are proven to be beneficial in one country and one specific dietary pattern, this does not mean that it is appropriate in other countries and contexts. Therefore, country-specific research is needed, but it is not possible for many countries to afford the costs associated with such research. The ultimate solution to promote health should be through healthy eating. Our decisions on eating should not be based purely on our understanding of the bioactive components. There are far more chemicals in food than what we know and treat as nutrients or bioactive components.

Based on the available evidence [1,16,17], it is wise to take caution towards the use of dietary supplements, especially for people with chronic conditions [5]. There is no evidence suggesting that people with a healthy dietary pattern can receive extra benefits from the use of dietary supplements. Dietary supplements should not and cannot be used as a method to treat the problems caused by an unhealthy diet.

## References

[1] Kim J., Choi J., Kwon S.Y., McEvoy J.W., Blaha M.J., Blumenthal R.S., Guallar E., Zhao D., Michos E.D. (2018). Association of Multivitamin and Mineral Supplementation and Risk of Cardiovascular Disease: A Systematic Review and Meta-Analysis. Circ. Cardiovasc. Qual. Outcomes.

[2] The Council for Responsible Nutrition (CRN) Dietary Supplement Usage Up Dramatically During Pandemic, New Ipsos-CRN Survey Shows [Press Release]. https://www.crnusa.org/newsroom/dietary-supplement-usage-dramatically-during-pandemic-new-ipsos-crn-survey-shows.

[3] Zhao A., Li Z., Ke Y., Huo S., Ma Y., Zhang Y., Zhang J., Ren Z. (2020). Dietary Diversity among Chinese Residents during the COVID-19 Outbreak and Its Associated Factors. Nutrients.

[4] Dwyer J.T., Coates P.M., Smith M.J. (2018). Dietary Supplements: Regulatory Challenges and Research Resources. Nutrients.

[5] Pandey A.C., Topol E.J. (2019). Dispense With Supplements for Improving Heart Outcomes. Ann. Intern. Med..

[6] Chen F., Du M., Blumberg J.B., Ho Chui K.K., Ruan M., Rogers G., Shan Z., Zeng L., Zhang F.F. (2019). Association Among Dietary Supplement Use, Nutrient Intake, and Mortality Among U.S. Adults: A Cohort Study. Ann. Intern. Med..

[7] Tachjian A., Maria V., Jahangir A. (2010). Use of herbal products and potential interactions in patients with cardiovascular diseases. J. Am. Coll. Cardiol..

[8] Alpha-Tocopherol, Beta Carotene Cancer Prevention Study Group (1994). The effect of vitamin E and beta carotene on the incidence of lung cancer and other cancers in male smokers. N. Engl. J. Med..

[9] Omenn G.S., Goodman G.E., Thornquist M.D., Balmes J., Cullen M.R., Glass A., Keogh J.P., Meyskens F.L., Valanis B., Williams J.H. (1996). Effects of a combination of beta carotene and vitamin A on lung cancer and cardiovascular disease. N. Engl. J. Med..

[10] Li M., Hu Y., Mao D., Wang R., Chen J., Li W., Yang X., Piao J., Yang L. (2017). Prevalence of Anemia among Chinese Rural Residents. Nutrients.

[11] Shi Z., El-Obeid T., Riley M., Li M., Page A., Liu J. (2019). High Chili Intake and Cognitive Function among 4582 Adults: An Open Cohort Study over 15 Years. Nutrients.

[12] Shi Z., Hu X., Yuan B., Pan X., Meyer H.E., Holmboe-Ottesen G. (2006). Association between serum ferritin, hemoglobin, iron intake, and diabetes in adults in Jiangsu, China. Diabetes Care.

[13] Li X., He T., Yu K., Lu Q., Alkasir R., Guo G., Xue Y. (2018). Markers of Iron Status Are Associated with Risk of Hyperuricemia among Chinese Adults: Nationwide Population-Based Study. Nutrients.

[14] Egan B., Hodgkins C., Shepherd R., Timotijevic L., Raats M. (2011). An overview of consumer attitudes and beliefs about plant food supplements. Food Funct..

[15] Garcia-Cazarin M.L., Wambogo E.A., Regan K.S., Davis C.D. (2014). Dietary supplement research portfolio at the NIH, 2009-2011. J. Nutr..

[16] Khan S.U., Khan M.U., Riaz H., Valavoor S., Zhao D., Vaughan L., Okunrintemi V., Riaz I.B., Khan M.S., Kaluski E. (2019). Effects of Nutritional Supplements and Dietary Interventions on Cardiovascular Outcomes: An Umbrella Review and Evidence Map. Ann. Intern. Med..

[17] Heravi A.S., Michos E.D. (2019). Vitamin D and Calcium Supplements: Helpful, Harmful, or Neutral for Cardiovascular Risk?. Methodist Debakey Cardiovasc. J..

